# The Intersectionality Toolbox: A Resource for Teaching and Applying an Intersectional Lens in Public Health

**DOI:** 10.3389/fpubh.2021.772301

**Published:** 2021-12-02

**Authors:** Natalie J. Sabik

**Affiliations:** Department of Health Studies, University of Rhode Island, Kingston, RI, United States

**Keywords:** intersectionality, critical analysis, teaching, public health, health disparities

## Abstract

Intersectionality is a theoretical framework that was developed to address the ways in which people's experiences are shaped based on their intersecting social identities (e. g., race/ethnicity, gender, class, age, etc.). This approach focuses on the importance of considering power, privilege, and social structures in relation to people's access to resources, experiences of discrimination, and interpersonal interactions. An intersectional approach in public health is critical for research and teaching to illuminate health disparities and the underlying structures that create and maintain disparities. While scholars have focused primarily on how to integrate an intersectional perspective into research methods, there is a need for a clear framework for applying intersectionality effectively in public health teaching. The Intersectionality Toolbox (ITB) is a framework developed from a variety of interdisciplinary resources designed to apply an intersectional perspective to public health issues. This article describes the Intersectionality Toolbox and details how it can be utilized in public health classes. Following a course where the ITB was implemented, student feedback was sought to determine the appropriateness and effectiveness of the design, and metrics were aligned with the learning outcomes. The ITB was refined and retained to integrate into courses and assignments focused on teaching about the intersecting nature of the social determinants of health.

## Introduction

The field of public health has a longstanding commitment to understanding and elucidating the social determinants of health (1). These factors represent individual and structural identities, characteristics, and patterns that shape health and well-being (2). However, the social determinants are not experienced in isolation; it is an individual's unique and intersecting position within social categories and structures, as well as their individual identities, that create the conditions in which they live. This position can be understood through the lens of intersectionality, a theoretical approach that helps to integrate individual and structural components of the determinants of health.

Intersectionality is a term that was created by critical race theorist Kimberlé Crenshaw in 1989 to illustrate how social identities were defined as isolated and mutually exclusive in legal scholarship. Crenshaw showed that when social categories and the associated identities (e.g., race/ethnicity, gender) were treated as mutually exclusive, the experiences of individuals who experience subordination based on multiple identities, such as Black women, were effectively erased (3). The term “intersectionality” has been widely adopted by researchers, academics, and the media to analyze the causes and effects of structural inequality (4, 5), and to highlight the embedded and pervasive inequalities underlying health disparities (6). In addition, scholars have called for the broader integration and examination of intersecting racial, ethnic, class, ability, age, sexuality and gender disparities (7).

An intersectional approach in public health is critical for research and teaching to illuminate health disparities and the underlying structures that create and maintain disparities (4, 8). Specifically, intersectionality can be seen as a lens through which students and researchers can investigate health issues that bring to light and make visible both individual experiences and how these are created by patterns of power, privilege, and the social structures and policies that contribute to inequality and a lack of health equity. While scholars have focused primarily on the challenges of integrating an intersectional perspective into research [e.g., (4, 6, 9, 10)], the field is currently in need of a framework for applying intersectional theory effectively in public health teaching.

The Intersectionality Toolbox is a framework developed from a variety of interdisciplinary resources designed to apply an intersectional perspective to public health issues. This article describes the Intersectional Toolbox and how to implement it in public health courses and includes the results of an assessment of an undergraduate course (Intersecting Social Identities and Health) in which the Toolbox was implemented. The interdisciplinary approach aimed to equip students with a concrete framework for understanding and investigating critical public health issues, and to prepare them to work effectively as future leaders in public health and related fields with knowledge of how to apply an intersectional lens to uncover and make visible social issues impacting public health.

The Intersectionality Toolbox was initially created as part of the Intersecting Social Identities and Health course developed as an elective to supplement the core curriculum for Health Studies majors. Students taking the course had previously completed an introductory public health course and were familiar with the basic tenets of public health, and foundational knowledge of public health is recommended prior to classes implementing the Toolbox. The Toolbox is re-created each time the course is taught by reviewing the current and relevant work on intersectionality in public health as well as related fields (e.g., psychology, gender studies, sociology, legal studies) and co-developing questions for analysis with the students in the course. The toolbox may vary each time it is constructed to reflect the current readings included in the course, and it is helpful for the instructor to have a sense of what major areas of inquiry or main questions will be included in the Toolbox. Through the discussion developing the questions the instructor can make suggestions, help refine language in the questions, and can ensure that a well-rounded set of questions is developed for analysis in the course. A sample list of the toolbox questions from the initial course is included in Table 1, along with the resources used to create the questions and further reading that complements and illustrates the genesis of each question. Instructors can use this list of readings and associated questions to guide the creation of a syllabus as well as discussion around the readings to draw out similar questions to populate the Toolbox. In addition, the Toolbox can be modified as needed to fit in existing public health courses by tailoring the readings and drawing relevant related questions to apply an intersectional lens to specific public health topics.

**Table 1:** Intersectionality toolbox questions, main concepts, and suggested readings.

**Question**	**Key concepts to apply**	**Suggested readings**
1. What are the shared social, historical, and cultural characteristics of a social group?	Understanding that social groups are not biologically or genetically bound; investigating the underlying social and cultural patterns that led to certain outcomes or disparities	(11–15)
2. Who is included and who is left out of this research?	Careful assessment of the demographic profiles of who is included in research; assessment of who has been made invisible or whose experiences are not represented	(16–18)
3. What are the critical differences defining groups experiences (as opposed to outcomes)?	Categorization of groups based on demographic or identity factors often leads to essentialization and a reduction to defining them solely as members of that group. An alternate approach asks what it is about being a member of a group that is important for this line of research?	(19–22)
4. Where is there variation within a particular group?	Looking at within-group variability rather than comparing across groups reveals nuanced information and reinforces the idea that groups are not homogeneous	(23–26)
5. Where are there similarities between groups?	In our quest to establish and track disparities, the focus is often on differences instead of similarities. Asking this question can reveal new and different information that would have otherwise been overlooked, and knowledge can be leveraged across groups.	(27–29)
6. What is the role of power, inequality, and oppression in understanding this issue?	This is often a subtext that is not clearly stated or addressed in health disparities research. However, this is foundational to understanding not only how health disparities are framed, but also how language is used to place the onus on certain groups to address the issues.	(30–32)
7. Are there other perspectives or angles to consider to the research beyond what was assessed?	Although this question may seem obvious, it's a good exercise to pause and consider (a) whose perspective is represented on the research team, (b) what factors or perspectives would enrich or potentially contribute to our understanding of a health issue or the population being studied. This is an invitation to go deeper and to search out nuance that is often missed in research based on mean comparison.	(4, 33–35)
8. What are the structural factors (laws, institutional practices, and policies) that impact someone's health?	Culturally in the US there is a focus on individualism and individual approaches to managing health. This question invites a contextualization of the individual into their broader social fabric, wherein the laws, institutions, social norms, and policies shape and constrain individual behavior. In addition, this question considers how policies are made and might be changed based on an intersectional perspective [see (36) for more].	(36–40)
9. What are the multiple social inequalities (e.g., racism, heterosexism, sexism, classism) that intersect to create and maintain health disparities?	A core tenet of intersectionality is that social identities are not experienced singularly, and people's experiences depend on the intersection of identities they hold. Dovetailing this approach is understanding that discrimination and power are also experienced in an intersectional nature. Direct acknowledgment of this complexity will allow for these patterns and structures to be made visible and acknowledged in producing and maintaining health disparities.	(41–44)
10. What can we learn by looking at social groups as social processes rather than characteristics of the individual?	Asking “what does it mean to be a member of a social group?” focuses on how that group membership is experienced by individuals in the group, rather than labeling the group and assuming what that membership means. This approach helps to center the experiences of people in minoritized groups and highlights information that may have otherwise been overlooked or assumed.	(23, 45–47)

In the course that implements the Intersectionality Toolbox, the students spend the first half of the course reading foundational works about the theory of intersectionality and drawing key questions and recommendations out of each reading to build a list of questions to serve as the Intersectionality Toolbox. Building the list of questions together in class is critical so that students actively co-create the material, are invested in understanding the material, and have time to process the underlying reasons for the questions and their significance. The course instructor can guide students toward any key questions that need to be added to adequately address the core concepts in the toolbox. The list of suggested readings can be expanded or amended with readings suited to the course, and additional questions or variations on the questions may emerge depending on the set of core readings presented. Once the students and instructor agree that the toolbox is complete, the second half of the course focuses on using the questions as a framework for analysis by selecting the most relevant questions and applying them by examining news stories and research about different populations and current health issues. Assignments require students to apply the learning by utilizing the toolbox questions to form their own analysis of health issues.

## Development of the Intersectionality Toolbox Questions

Table 1 summarizes a set of sample questions in the Intersectionality Toolbox and provides a brief description of the main concepts associated with each question. In addition, references for further reading that elaborate on the questions and key concepts are included. The associated readings can be used in the course to structure discussion and to provide further context for each of the toolbox questions. As new scholarship and examples related to each question emerge, teachers and researchers can adapt or update this framework to reflect current advances in the field. In addition, if a course focuses on a specific topic or issue within the field of public health, core intersectionality readings could make up a smaller portion of the overall course to build a list of questions for analysis to apply to the issue or topic of focus for the course.

In the initial class that implemented the ITB, the instructor and students began with an empty Intersectionality Toolbox slide, and discussion was based around identifying and refining the questions for the toolbox. One suggestion to assist the students in drawing out the key questions is to ask students to consider “what questions or suggestions emerge from this reading that we can add to our Toolbox?” while they are reading each article. Many of the sample papers included in Table 1 make specific recommendations and/or raise specific questions that the class can draw on directly to populate the toolbox, and often provide examples or detailed description of how and why these questions are needed for an intersectional analysis.

When the class has agreed on how to frame a question, the question is added to the list, which is populated over the first half of the class. If needed, the instructor can guide the development by making suggestions or additions to ensure that the list thoroughly addresses the major aspects of intersectionality. The focus during this section of the course is to read and discuss readings on the foundations of intersectionality and to note what questions, suggestions, or recommendations arise from these readings.

## Application of the Intersectionality Toolbox Questions

Following the development of the Intersectionality Toolbox (ITB) questions, students utilize the questions to analyze current health issues and topics. The questions are applied through two major assignments that build on each other to provide students with practice and to provide feedback on the application of the ITB questions to current health issues. First, students complete a news article analysis report. For this assignment, students select a current news story (e.g., reporting in a reputable newspaper or news source) that focuses broadly on an issue related to health. Students perform an analysis of the news story based on the ITB questions; typically, students choose 3–5 questions that are most applicable from the list and critically evaluate the information from the news source. Students prepare a written response articulating their analysis (~2–3 pages), as well as a 5-min verbal presentation that allowed them to share their insights with the class. This approach can be modified depending on the class size, though it is critical that students receive feedback on this assignment to further develop their intersectional analysis. During the in-class presentations, allocating 5–10 min for questions and follow up discussion allows for the exploration of any ITB questions not covered in the presentation that would also apply to the topic, and any additional information or analysis that might be noted by other students or the instructor. This assignment is well-suited to be a group activity, and if it is appropriate based on the size and time available in the course, the instructor can form groups of 3–4 students to conduct this analysis and present the material together. This assignment allows for students to test out ITB questions and answers together and creates an opportunity for students to discuss the application of the ITB questions in a small group context.

The active feedback approach helps students practice these skills in a low-stakes assignment and models for everyone in the class how the ITB questions can actively be applied. This practice is iterative and requires the development of critical thinking and communication skills. Further, this practice helps students' thinking evolve so that they are well-prepared to conduct a more in-depth and critical analysis for their final project.

For the final project students are instructed to perform an intersectional analysis of a current health issue. First, students choose a current health issue that is represented as a health disparity. Utilizing their research skills, students must address the questions: who is disproportionately affected by this health issue? And what studies have been done that look at different identities/groups regarding this health issue? Second, students identify who has been included and who has been left out when examining research on this health issue. In addition, students consider the following questions: has any research been conducted that takes an intersectional perspective? If so, what did this reveal about the health issue? Third, students perform an intersectional assessment of the health issue. Using the readings and questions from class, students must reflect on the research about the health topic from an intersectional perspective and apply 3–5 questions from the ITB to the issue.

Last, students must make recommendations informed by their research about how to enhance this area of research. Students are asked to make specific recommendations about how a researcher or journalist might consider the health issue from an intersectional perspective. To do so, they must address what other factors need to be considered, and why? How might groups that have been overlooked be affected by this health issue? In this section, students should make specific suggestions about how students and researchers in public health can broaden the understanding of the health issue by applying intersectional knowledge.

### Setting and Students

The course was first offered from January to April 2018 at the University of Rhode Island to current health studies undergraduate students. The course was subsequently offered from January to April 2020, to the same population of students. During the 2020 course offering, the course unexpectedly moved online in response to safety concerns related to COVID-19; at that time, all aspects of the course were transitioned to be delivered remotely. The demographics of the students in the course were reflective of the demographics of the major, with the majority of students reporting they were from Rhode Island (62%), identified as White (60%), and identify as women (80%).

Students were required to have previously completed core courses for the major, including an Introduction to Public Health course and an intermediate level course on Interdisciplinary Approaches to Public Health to ensure that basic concepts and competencies were adequately addressed prior to beginning this course. Due to the prerequisite requirements, the course is listed as an upper-level elective and is targeted at advanced undergraduates who are typically of junior or senior standing.

### Course Evaluation

Evaluations for the course were delivered using an online survey that students completed at the end of the course. Two types of evaluations were collected. First, the University initiated an assessment of learning outcomes via self-reported quantitative assessments. Second, the instructor collected self-reported qualitative data to assess the effectiveness of the course and student engagement. Data were gathered evaluating the course in 2018 and 18 of the 28 students enrolled in the course completed the quantitative assessment. Due to the pandemic conditions in 2020, the university opted not to gather course evaluation data. The quantitative assessments were based on asking students to rate how much progress they made on each outcome on a 5-point scale ranging from 1 = no apparent progress to 5 = exceptional progress. Higher scores indicate greater progress toward course objectives.

The surveys generated self-reported qualitative and quantitative findings reflecting the effectiveness of the course in meeting student learning outcomes. Data were analyzed and reflected the effectiveness of the teaching tool and the course. In addition, student scores on the final assignment integrating the ITB with current research on health issues reflect the student's ability to effectively apply the material.

Representative quantitative data on the most relevant learning outcomes are presented in Table 2. In addition, student scores on the final project reflect their ability to integrate the information and apply it to current health issues. The assignment and grading rubric are presented in the supplementary materials, and evaluations of student work are presented in the Table 3.

**Table 2:** Students 2018 assessment of progress on learning outcomes.

**Question**	**Mean response**	**1**	**2**	**3**	**4**	**5**
1. Gaining a basic understanding of the subject (e.g., factual knowledge, methods, principles, generalizations, theories).	4.5/5	0	0	11	28	61
2. Developing knowledge and understanding of diverse perspectives, global awareness, and other cultures.	4.6/5	0	0	0	39	61
3. Learning to apply course material (to improve thinking, problem solving, and decisions)	4.4/5	0	0	11	39	50
4. Developing specific skills, competencies, and points of view needed by professionals in the field most closely related to this course.	4.4/5	0	0	6	44	50
5. Learning how to find, evaluate, and use resources to explore a topic in depth.	4.4/5	0	0	17	22	61
6. Learning to analyze and critically evaluate ideas, arguments, and points of view.	4.3/5	0	0	17	33	50
7. Learning to apply knowledge and skills to benefit others or serve the public good.	4.4/5	0	0	11	33	56

*Questions were asked following the phrase “describe your progress on…” Answer categories were: 1, no apparent progress; 2, slight progress; 3, moderate progress; 4, substantial progress; 5, exceptional progress*.

**Table 3:** Assessment of student work reflecting the integration of ITB knowledge.

**Assessment %**	**Percent of students (2018, *n* = 25)**	**Percent of student (2020, *n* = 26)**
70–80	8% (*n* = 2)	4% (*n* = 1)
80–90	40% (*n* = 10)	19% (*n* = 5)
90% and above	52% (*n* = 13)	77% (*n* = 20)

## Results

### Quantitative Findings

Findings from the quantitative items on student surveys indicated that, on average, students noted between substantial (a score of 4/5) to exceptional (a score of 5/5) progress on all learning outcomes. Students reported a significant increase in gaining factual knowledge about the subject, developing knowledge, understanding diverse perspectives, applying course material, developing competencies and skills needed in the profession, learning to find and evaluate resources, critically evaluating points of view, and applying knowledge to benefit others and serve the public good. Many of these outcomes are aligned with the broader aims of public health and student data indicated that the approach in the course was effective at building the skills and knowledge to apply intersectionality to the field.

Student scores on the final assignment were used to gauge mastery over the concepts from the toolbox and application of the questions to current health issues. Overall, scores indicated their familiar with the toolbox principles and ability to effectively analyze a current health issue using this framework for their analysis. The majority of students in both classes earned a 90% or higher on the assignment, demonstrating the student's ability to integrate the work and apply it to real-world health issues.

### Qualitative Findings

Open-ended questions eliciting students' comments and feedback on the course indicated that the course was very positively received. When asked what students would change about the course, many said “nothing” or indicated that they thought the overall course structure and content were well-designed. A few students noted that the readings were challenging, and that having both discussion and reading guides to walk through this work was beneficial and should be retained. Reading guides are a structured set of questions designed by the instructor to accompany each course reading and the questions help students focus on the main ideas and most critical questions and content in each reading. Responses indicated that these were generally well-received and helpful in breaking down more complex ideas. In addition, several students reported that having time in class to walk through examples of how to apply the ITB questions was very helpful, and many commented that they benefited from an assignment asking students to choose a current news article about a health issue and apply a few questions from the ITB. This hand-on assignment illustrated how to work through the analysis using the ITB questions in a low-stakes and mutually constitutive assignment prior to the individual final assignment. Feedback from the 2018 course indicated that the structure worked well for nearly all students, and all major course components were retained.

## Discussion

The Intersectionality Toolbox represents a framework for teaching students how to apply complex and multi-level thinking to critical public health issues. The objective of using the Toolbox is to guide students through the development of the questions so that they understand the foundations of intersectionality theory and are empowered to apply the questions in a tangible way. The findings indicate that after taking the course, most students not only grasped the core concepts but were able to independently apply them to current health issues and trends. The course feedback serves as a promising indication that students are learning to identify and address complex social issues that determine the health of community populations as they enter the workforce or pursue further education.

The implications for the development of the ITB are significant for public health education, as this framework serves as a basis for further development and refinement of the application of intersectionality theory as new and updated scholarship emerges. The ITB is designed to be flexible and to be tailored to public health courses looking to include intersectionality theory into the classroom. Given the focus in public health on addressing health disparities and drawing on the social determinants of health, this framework is emerging as a critical nexus for nuanced approaches to core issues in the field. Further, students need to be well-versed in current approaches in the field, and intersectionality theory is becoming more visible and widely adopted in public health; integrating this tool into existing curriculum will strengthen the pedagogical approaches that seek to understand the complexity of current health issues and will equip students with the vocabulary and knowledge to apply an intersectional approach in their future work. Public health educators may move toward considering a revision of student learning outcomes that include this framework and utilizing this tool as a concrete way to implement intersectionality in the classroom is a necessary first step. In the long term, public health educators will need to integrate intersectional theory into their teaching, and the preliminary results from the findings presented in the paper suggest that utilizing the ITB framework is an effective approach to begin this process.

The data from the class have several limitations that need to be considered. First, the evaluations were primarily self-reported by students and were drawn from a university-wide assessment used to determine progress on learning outcomes. In future iterations of the course, more specific assessments tailored to the ITB and the effectiveness of applying intersectionality theory are needed. More nuanced and specific questions regarding students' experiences in the course and with the material will provide greater insight into the implementation and refinement of the ITB. Second, not all students elected to complete the course evaluation. A strong effort is made to gather this data, including time dedicated to allowing students to complete the assessment as well as numerous reminders in class and via email message to encourage participation. However, it is ultimately up to the students to determine whether they want to provide feedback on the course. The number of evaluations completed is in line with other courses offered in the department. Last, to protect the anonymity of the students in the course, individual level demographic information from the students was not collected. Although an overview of the student population was provided in the method, future research on the effectiveness of the ITB may be strengthened by the inclusion of individual-level demographic data linked to course outcomes if student anonymity can be maintained.

## Conclusion

A clear framework for understanding and applying an intersectional perspective is urgently needed to address health equity and to guide students through a process wherein they learn to (a) see the interlocking nature of the social determinants of health, (b) reconcile social determinants with individual's identities and experiences, (c) identify the structural factors that influence health (e.g., public policy, institutional policies, and cultural norms and stereotypes), and (d) analyze complex health issues by engaging in multi-level thinking. One aim of public health is to end health disparities and to increase health equity, but this requires a shift in perspective that encourages a deeper understanding of the causes and consequences of current health issues. The Intersectionality Toolbox is an approach that equips students with the knowledge and skills to apply broadly to their work both in and beyond the classroom.

## Data Availability Statement

The original contributions presented in the study are included in the article/supplementary material, further inquiries can be directed to the corresponding author.

## Author Contributions

The author confirms being the sole contributor of this work and has approved it for publication.

## Conflict of Interest

The author declares that the research was conducted in the absence of any commercial or financial relationships that could be construed as a potential conflict of interest.

## Publisher's Note

All claims expressed in this article are solely those of the authors and do not necessarily represent those of their affiliated organizations, or those of the publisher, the editors and the reviewers. Any product that may be evaluated in this article, or claim that may be made by its manufacturer, is not guaranteed or endorsed by the publisher.
